# Preliminary evidences of the presence of extracellular DNA single stranded forms in soil

**DOI:** 10.1371/journal.pone.0227296

**Published:** 2020-01-07

**Authors:** Shamina Imran Pathan, Paola Arfaioli, Maria Teresa Ceccherini, Judith Ascher-Jenull, Giacomo Pietramellara

**Affiliations:** 1 Department of Agri-food, Environmental, Forestry Science and Technology (DAGRI), University of Florence, Piazzale delle Cascine, Florence, Italy; 2 Institute of Microbiology, University of Innsbruck, Innsbruck, Austria; Free University of Bozen/Bolzano, ITALY

## Abstract

The relevance of extracellular DNA (eDNA) in the soil ecosystem is becoming more and more evident to the scientific community by the progressive discovery of functions accompanying to natural gene transformation. However, despite the increased number of published articles dedicated to eDNA in soil, so far only few are focused on its single stranded form (eDNAss). The present paper is the first to investigate the quantitative relevance of eDNAss in the total soil eDNA pool, discriminating between its linear (eDNAssl) and circular (eDNAssc) forms and the respective weakly (*wa*) and tightly (*ta*) adsorbed fractions. The results showed the prevalence of eDNAss and its linear form in both the total soil eDNA pool and its *wa* and *ta* fractions. Both of the eDNAss fractions (linear and circular) were characterized by small fragments.

## Introduction

Extracellular DNA (eDNA) can represent up to 40% of the total soil DNA pool [1,2]. Despite the interest of the scientific community on eDNA in soil is quite recent, the studies have highlighted several additional ecological functions to genetic exchange through natural transformation [3]. In fact, in relation to the soil bacterial community, eDNA can act as a source of nutrients [4, 5, 6], as constituent of biofilms [7, 8, 9] and bio-crusts [10], as agent to influence the porosity of soil aggregates [11, 12], and as signaling- or chemoattractant molecule [13, 14, 7]. For plants, the soil eDNA can act as source of nutrients [15], as constitutive component of roots extracellular traps defense against biotic [16] and abiotic [17] hazards, as hormone simulant [15, 18] and allelopathic [18] molecules.

All these functions and peculiar traits make eDNA to one of the most interesting molecules in soil, capable to affect both the composition and activity of microbial and plant communities. However, despite the increasing evidences on multiple roles and functions of eDNA in soil, the discrimination between its single and double stranded fractions has been up to now neglected although they are characterized by different reactivity and adsorption capacity [19, 20, 21]. To the best of our knowledge, there are only few studies dealing with eDNAss with focus on its adsorption onto clays [19, 21] and other minerals [22] under laboratory conditions. Thus, the present study aimed to assess the most mobile fraction of soil eDNA, based on the sequential extraction method by Ascher et al. [1], that is capable to specifically evaluate and characterize the eDNA fractions that are weakly (adsorbed) and strongly bound onto soil colloids, thus discriminating between the DNA forms with high and low mobility in soil.

The experimental approaches tested were an indirect and a direct method, to improve the discriminatory assessment of the single stranded (ss) and double stranded (ds) DNA forms. The proposed indirect approach is an optimization of the method by Gardner and Gunsch [21] that originally discriminated between the extracellular DNA fractions, ds and ssDNA, via subtraction of the amount of dsDNA (determined by Qubit using the DNA intercalating fluorochrome PicoGreen) from the total DNA (determined by Nanodrop UV-Vis spectrophotometer). Our modifications are based on the assumption that the soil total eDNA pool represents a mix of ds and ss forms within a wide range of different molecular sizes (molecular weight). Compared to the indirect approach, our proposed direct approach provides more precise information about eDNAss by discriminating between circular and linear forms, that can be further characterized in detail via downstream analysis.

The present paper is a first attempt to investigate in-depth on eDNAss in soil with the objectives to i) quantitatively assess the eDNAss as well as to discriminate between its linear (eDNAssl) and circular (eDNAssc) forms; ii) avoid bias in the quantification of eDNAss; and iii) define the adsorption strength of the eDNAss linear and circular fractions.

## Material and methods

The sampling site, named Cavalla, is located in Vallombrosa forest at Tuscany Appennine 50 km east of Florence, Italy with an extension of 13 km^2^ at an altitude between 600–1300 m above sea level. Mean annual temperature and precipitation ranging from 8–12 ºC and 1000–25000 mm, respectively. The soil is classified as a fragic distrudept soil (Table 1) with Silver fir (*Abies alba Mill*.) and European beech (*Fagus sylvatica L*.) as dominant trees [23]. Samples were collected from public land and permits were not required as local people could have access to this site and could collect different forest products except forest animals. Further, soil sampling is permitted for research in which protected or threatened species or locations are not involved, such as in our case. Moreover, we confirm that no protected or threatened species or locations were involved in the study.

**Table 1:** Selected properties of the A1 and A2 horizons of the study soil (Vallombrosa, Italy).

Horizon	Depth (cm)	pH (H_2_O)		pH (KCl)		Org. (C g.kg^-1^)	Total N (g.kg^-1^)	BS%	Sand %	Clay
**A1**	5.7 (1.2)		4.5	3.5		36 (12)	5.4 (0.0)	42(12)	34 (8)	21 86)
**A2**	28.3 (0.5)		4.6	3.7		17 (5)	2.0 (0.0)	26 (13)	33 (10)	22 (6)
	**Structure**		**The main cations present in the soil solution**							
**A1**	medium crumbs		Mg^2+^ and Ca^2+^							
**A2**	blocky sub-angular		Mg^2+^ and Ca^2+^							
**Mineralogical composition of sandstone bedrock termed macigno**										
**Quarz**										
**Plagioclases**										
**Phyllosilicates**	Chlorite	Calcite		Kaolinite		Micas	Vermiculite			

Number in parentheses are the standard errors (n = 3)

**Fig 1:**
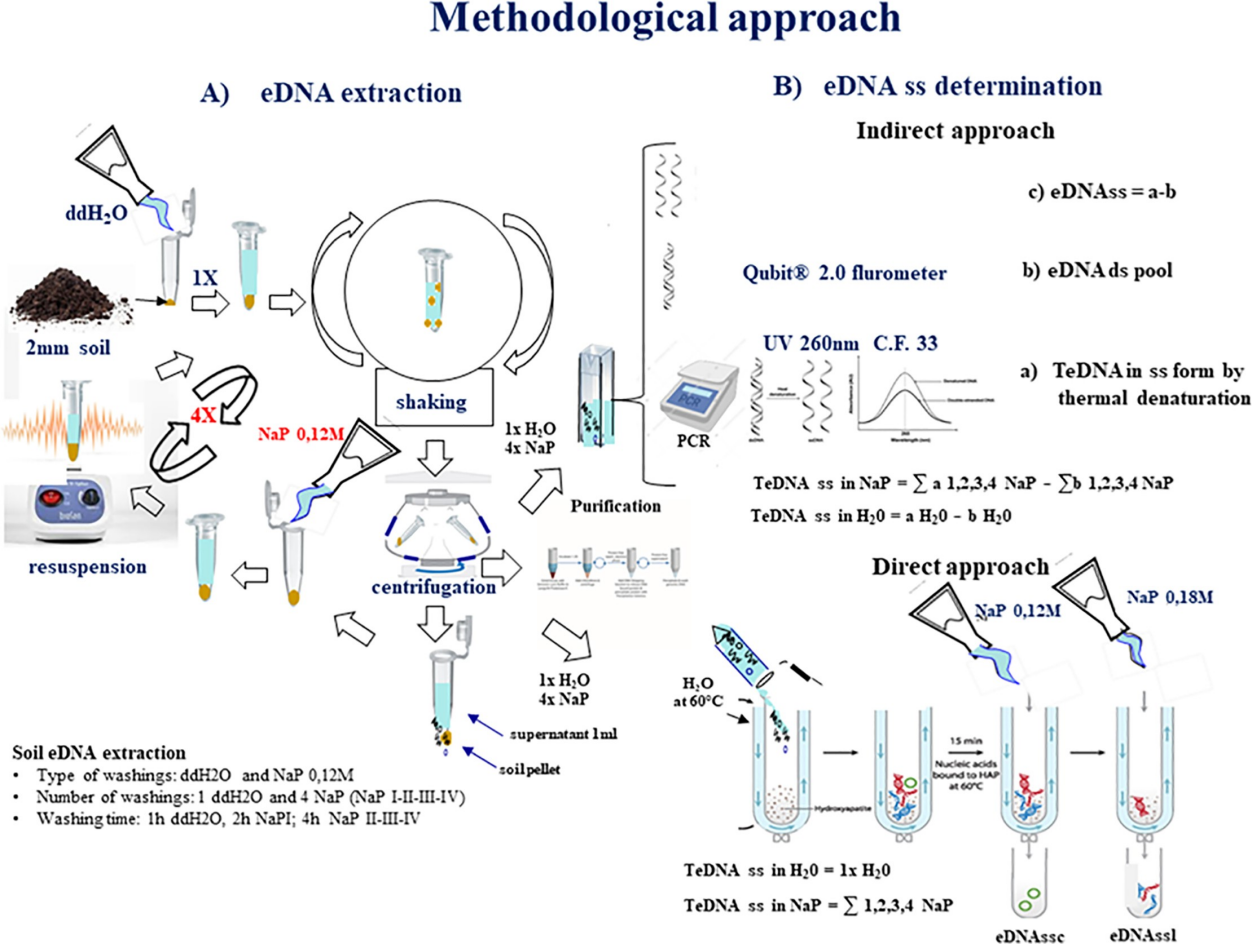
Schematic illustration of the experimental method. eDNA extracellular DNA; ss single stranded DNA; ds double stranded DNA.

The sampling area, Cavalla, cover an area of 1200 m^2^ and is characterised by high soil, vegetable cover, slope and exposure homogeneity. The soil was sampled in May 2018 to a depth of 10 cm (includes horizon A1 and part of A2 horizon), air dried, sieved at 2 mm and stored at -20°C prior to further analyses. Soil samples (one kg) were collected randomly from three different sampling points. The extraction of the weakly (*wa*) and tightly (*ta*) eDNA fractions from the three field replicates was done by sequential elution with deionized distilled water (ddH_2_O) and Na_2_HPO_4_ (NaP) 0.12M, respectively [24]. With the intent to increase the yields of extractable eDNA from soil, the number and duration of washing cycles were scheduled on the base of our experience on Vallombrosa soil from previous studies. In detail, the duration of the elution washing cycles for the different eDNA fractions was extended from 30 min to 1 h (ddH_2_O) for the weekly bound (*wa*) fraction, and from 30 min to 2 h (NaPI) and 4 h for the first one and for each of the subsequent three washings (NaPII, NaPIII, NaPIV) for the tightly bound (*ta*) fractions. The number of elution washings was increased only for the extraction of eDNA*ta* from one to three washings (NaPII, NaPIII, NaPIV). The total number of samples was 15 including replications (3 –eDNA*wa* and 12 –eDNA*ta*). All of the eDNA fractions were finally purified as described by Ascher et al. [1] (Fig 1A). The detection of the eDNA ds and ss forms in the *wa* and *ta* fractions was performed by the indirect approach, whereas their additional discrimination into the linear and circular forms was obtained by the direct approach as illustrated in Fig 1B.

### Indirect approach

The amount of soil eDNAds was quantified by Qubit® 2.0 fluorimeter in both *wa* and *ta* fractions. The quantification of eDNAss was performed by UV-Vis spectrophotometer (PicoDrop 260 nm), modifying the method of Gardner and Gunsch [21] by assuming that the total soil eDNA pool is a mix of differently sized ds and ss forms. Thus, its spectrophotometric quantification is biased by the utilized conversion factor (CF); CF 50 for DNAds and CF 33 for DNAss. To overcome this bias, the total amount of eDNA was thermally denatured into its single stranded form by PCR treatment at 94°C for 20 minutes [25] and then spectrophotometrically determined by utilizing the DNAss specific CF 33 (according to the manufacturer’s manual). To avoid the re-association process, the total eDNAss samples were stored on ice and frequently checked by Qubit® 2.0 fluorimeter and, if detected, converted in ss forms by the equation:

(DNAmeasure/50)x33=DNAss(μg/gdrysoil) (1)

The amount of eDNAss was then calculated by subtracting the amount of eDNAds (Qubit) from the total eDNA denatured into the ss form. The molecular weights of the eDNA *wa* and *ta* fractions were determined by agarose gel electrophoresis (1x Tris Acetate-EDTA buffer; 1:10,000 EtBr; 0.8% w/v; 100 V 60 min) in comparison to a DNA Mass Ladder Mix (Fermentas, 75 bp–20 Kb), without discriminating between its ss and ds forms (S1A Fig).

### Direct approach

We tested also a direct approach to discriminate between the eDNAss linear (eDNAssl) and circular (eDNAssc) forms by Ca^2+^-Hydroxyapatite (HAP) chromatography [26] through elution of purified eDNA *wa* and *ta* samples (Fig 1B). The approach is based on the different interaction strengths of the eDNA molecules with Ca^2+^ ions on the surface of HAP in relation to the number of involved phosphate groups that depends on its molecular size and conformation. Thus, the sequential washing of the Ca^2+^-HAP column with NaP buffer 0,12M and 0,18M is capable to elute the eDNAss circular and linear forms, respectively. The obtained eDNAss *wa* and *ta* circular (eDNAss*wa*c, DNAss*ta*c) and linear (eDNAss*wa*l, eDNAss*ta*l) forms were then quantified at 260 nm by PicoDrop spectrophotometer by utilizing the DNAss specific CF 33.

The eDNAss*wa* and eDNAss*ta* forms were also qualitatively characterized by agarose gel electrophoresis (1× Tris Acetate-EDTA buffer; 1:10,000 EtBr; 1,2% w/v; 100 V 60 min with a DNA Mass Ladder Mix Fermentas 0,1–10 Kb) and quantified by PicoDrop spectrophotometer by utilizing the DNAss specific CF 33 (S1B Fig).

### Statistical analysis

Two-way analysis of variance (ANOVA, extraction time x DNA form) was applied to check for any significant effect of extraction time, DNA ds and ss forms and their interaction on the variability of the eDNA yield. Further, a multiple pairwise comparison of means was done by Tukey’s HSD (honestly significant difference) test at P <0.05 level of significance, to assess individual effects of each factor. Statistical data processing was done using Past 3.06 [27].

## Results

**Fig 2:**
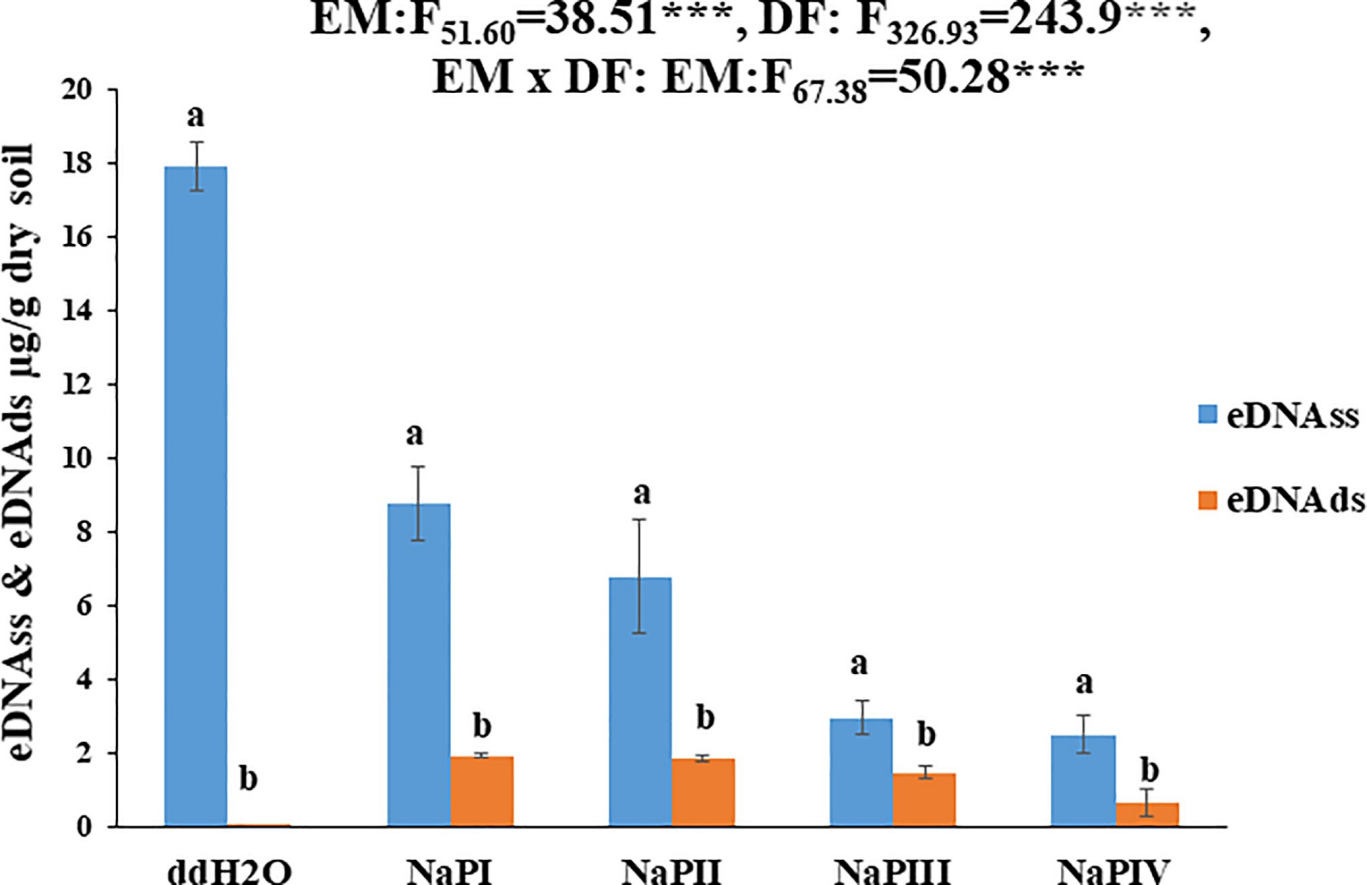
Yields of single stranded extracellular DNA (eDNAss) and double stranded extracellular DNA (eDNAds) expressed as eDNA (μg/g dry soil) extracted by using sterile water (ddH2O) and alkaline Sodium phosphate buffer (NaP) for different durations (hours) (NaPI = 2h, NaPII = 4h, NaPIII = 8h, NaPIV = 12h). Bars represent mean values with standard errors (n = 3); the letter indicates significant differences between ss and ds DNA concentrations. Further two-way ANOVA was performed to assess the effects of the extraction method (EM), DNA form (DF) and their interactions.

The indirect approach is suggested to be capable of i) determining the soil eDNAds and eDNAss pool and ii) discriminating their *wa* (ddH_2_O) and *ta* (NaPI, NaPII, NaPIII, NaPIV) fractions (Fig 2). Furthermore, the total eDNAss desorption quantification by the original indirect approach [21] evidenced a significant overestimation of total eDNAss amount [21]. The observed overestimation is most probably due to the utilization of the CF 50 in the UV-Vis quantification that is only accurate for the DNAds (Table 2) (see Materials and Methods).

**Table 2:** Yields of single stranded DNA (μg/g dry soil) extracted by ddH_2_O and alkaline NaP buffer by sequential washings and measured by comparing the method by Gardner and Gunsch (2017) Fig 1] and our proposed methods. The statistically significant differences between the different extraction methods are shown in different letters.

	Picodrop ds50-qubit	Single strand by denaturation at 94°C	DNA read as directly ss33	Total single strand ss33 by Ca-HAP
**ddH_2_O**	27.47±1.88^a^	19.45±0.38	17.92±0.66	2.22±0.31^a^
**NaP I**	16.37±2.86^b^	9.99±0.2	8.77±1	5.71±0.52^b^
**NaP II**	7.7±1.11^c^	5.6±0.24	6.79±1.54	4.69±0.33^b^
**NaP III**	2.26±0.59^c^	1.68±0.06	2.96±0.47	4.22±0.60^b^
**NaP IV**	2.7±0.38^c^	1.93±0.41	2.49±0.51	3.64±0.22^b^

**Fig 3:**
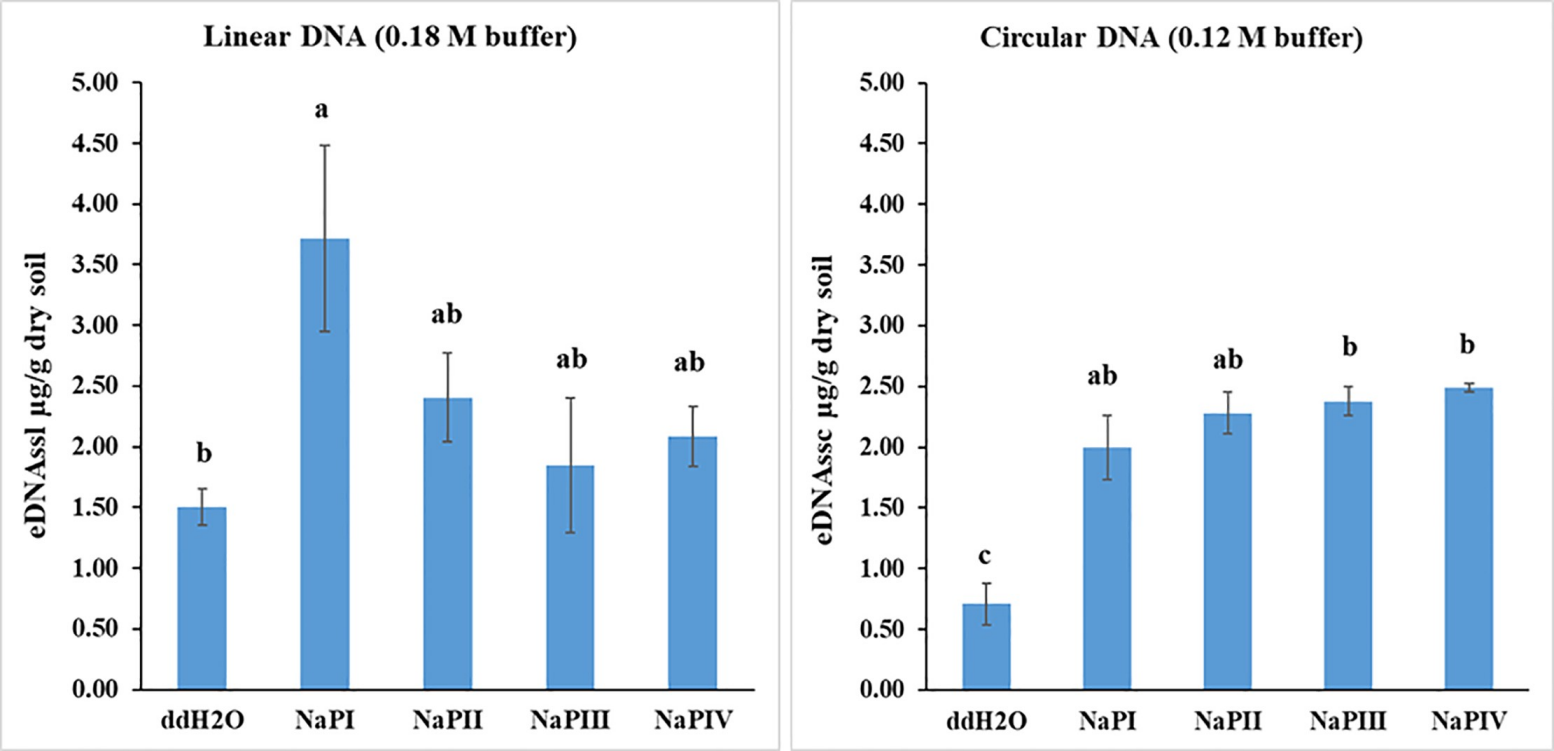
Yields of single stranded extracellular DNA (eDNAss μg/g dry soil) extracted by HAP chromatography using sterile water (ddH2O) for the weakly adsorbed fraction (eDNAsswa) and alkaline Sodium phosphate buffer (NaP) for the tightly adsorbed/bound fraction (eDNAssta) for different durations (hours) (NaPI = 2h, NaPII = 4h, NaPIII = 8h, NaPIV = 12h, only for the tightly adsorbed/bound fraction). Bars represent mean values with standard error (n = 3); the letter indicates significant difference between different extraction methods and duration.

The results of two factorial ANOVA analysis of the modified indirect approach have confirmed that the extraction steps (time) and DNA conformation significantly affected the yields of eDNA, both in its single stranded and double stranded form (Fig 2). Furthermore, the one-way ANOVA (pair wise comparison) results showed that the eDNAss concentration was significantly higher compared to eDNAds for both *wa* (ddH_2_O) and *ta* (NaPI, NaPII, NaPIII, NaPIV) fractions (Fig 2). The proposed direct approach has successfully detected the presence of eDNAss, linear and circular forms (Fig 3), highlighting the prevalence of eDNAssl in the eDNAss*wa* (ddH_2_O) and eDNAss*ta* (NaPI, NaPII, NaPIII, NaPIV) fractions. The relevance of the linear form in the eDNAss*ta* fraction is attributable to the NaPI washing with high extraction yield, whereas the subsequent three washings (NaPII, NaPIII, NaPIV) have shown the progressive increase in the quantitative relevance of the circular form (Fig 3). Regarding the detection of total eDNAss*wa*, it has to be pointed out that the significant lower detection of eDNAss*wa* was obtained by the direct approach compared to the indirect one. This discrepancy casts also doubt on the data obtained with respect to the linear and circular fractions of eDNAss*wa*.

The one-way ANOVA analysis of the results of the direct approach revealed significant differences only as a function of the desorbing agent (Fig 3). Furthermore, the qualitative characterization of the eDNA ds and ss forms by agarose gel electrophoresis evidenced a higher fragment size (molecular weight) for eDNAds with respect to eDNAss (S1A and S1B Fig). The agarose gel of eDNAss revealed its extremely small molecular size, as reflected by the presence of a smear in correspondence to the minimum fragment size of the utilized DNA Mass Ladder (S1B Fig).

## Discussion

The results of our proposed comparative approach, although being preliminary, revealed quantitatively relevant amounts of eDNAss in the soil eDNA pool (Fig 2), thus, providing the very first evidence of an important ecological aspect related to the eDNA degradation in soil. In fact, the experimental evidences on the dominance of eDNAss in the total soil eDNA pool combined with its extremely small molecular size (S1B Fig) and the absence of the eDNAds forms in the eDNA*wa* fraction (Fig 2) indicated that eDNAss results from the final step of the degradation process of eDNA in soil. Furthermore, based on the detected prevalence of DNAss in the eDNA*wa* fraction, DNAss can be clearly defined as the dominant form of the mobile soil eDNA pool. These results were confirmed by both the direct and indirect approach, thus, further supporting our findings.

In addition, both the properties of the eDNAss molecules and those of the studied soil (Table 1) further provide a plausible explanation of the obtained results [28]. First, the detected dominance of Mg^2+^ and Ca^2+^ cations in the soil solution (Table 1) have limited the interaction of the phosphate groups (strong) of eDNAds and eDNAss with the -O(H), -Si, -Al, -Fe sites of the soil minerals [29, 19], and stimulated their electrostatic interactions (weak) [30, 31]. Furthermore, the small molecular size of eDNAss (S1B Fig) has favored its adsorption in high amounts but also limited the binding sites per molecule, thus, facilitating its high desorption as evidenced by the high amounts of both *wa* and *ta* fractions (Fig 2) [32]. Also the prevalent hydrophilic characteristics of the acid mull humus form present in the sampled soil [23] might have reduced the strength of their bonds with the adsorbed eDNAss by limiting the contribution of its hydrophobic N bases and thus, facilitating its desorption [33].

The high fragmentation observed for eDNAss was probably due to its biotic degradation as supported by the high microbial activity [34] and phosphoesterases activitiy [23] in the soil. Despite a pending in-depth discussion of our findings due to the lack of related research, an additional explanation of the degradative processes of eDNA might also come from the activity of earthworms burrowing, reported for the studied soil [23], through their gut’s microbial community. It is also relevant to underline that the small molecular size of the adsorbed eDNAss fragments has drastically limited the degradative efficiency of microbes and extracellular enzymes [35, 36], thus promoting their persistence and a potential accumulation in soil over time [37].

Regarding the eDNAssl and eDNAssc *ta* fractions, their similar desorption values (Fig 3) contradict, which might be due to the soil pH. In fact, the soil sub-acid pH (5.0) value, correspondent to the isoelectric point of DNAds [38], should have induced a positive charge on the N-base of the eDNAss molecule by protonation, thus differentiating the molecular reactivity of its linear and circular forms. Furthermore, the detected prevalence of eDNAssl in both the eDNAss*wa* and eDNAss*ta* fractions (Fig 3) is of ecological relevance, considering the dominance of the circular form in nature [39]. Moreover, the higher yield of extracted total eDNAss by the direct approach in the last two NaP washing cycles (III-IV) suggests a higher protection rate of the eDNAss fractions.

Finally, part of the desorbed eDNAss*ta* potentially represents detached fragments of partially degraded large double stranded molecules, previously strongly adsorbed onto soil components [35, 36,40].

Importantly, the proposed direct and indirect approach highlighted two peculiarities:

- The overestimation of the amounts of desorbed total eDNAss by the original indirect approach [21] with respect to our proposed modified version, that is ascribable to the selected CF for the DNA quantification by UV-Vis spectrometry (see Materials and Methods); thus, our modified method provides an optimization of the method by Gardner and Gunsch (2017) [21], yielding reliable quantitative results;
- The lower total amount of eDNAss*wa* desorbed from soil detected by the direct approach with respect to the indirect approach can be ascribed to the elution of the smaller DNAss fragments by Ca-HAP chromatography and the high reactivity of the nucleotides at their molecular ends (NTE). In fact, the two free hydroxyl groups of the NETs' phosphate significantly increase the bonding strength on Ca-HAP for small DNA molecules.

With respect to our research hypotheses, the present research provided evidences on the quantitatively relevant presence of single stranded forms in the soil eDNA pool, discriminating also between its circular and linear fractions. This approach overcomes methodological restrictions, thus improving the potential of an in-depth assessment of the eDNA degradation processes in soil [41], basic for its ecological relevance in terms of integrity [24], persistence [37] and mobility [42]. In the light of these results, it is relevant to consider that some of the published studies on eDNA in soil [21, 24, 34, 40] have elaborated data and, consequently, interpreted findings potentially biased also by the presence of relevant amounts of eDNAss. Finally, although the eDNAss*wa* seems to be affected by a bias, the proposed (optimized) indirect and direct approaches are suggested to provide a powerful tool for an in-depth assessment of the extracellular soil DNA pool, capable to also discriminate between the circular and linear forms of the eDNAss molecules, contributing thus to a more correct interpretation of the genetic information deriving from soil DNA based downstream analysis.

## Supporting information

S1 Fig Data availability statement-supporting information. (DOCX)
