# Supplementary material for: Preliminary evidences of the presence of extracellular DNA single stranded forms in soil
Source: PLoS One. 2020 Jan 7;15(1):e0227296. doi: 10.1371/journal.pone.0227296 (PMC6946138; doi:10.1371/journal.pone.0227296)

Supplementary Figure 1. Characterization and fragment length distribution of different forms of extracellular DNA (eDNA) by agarose gel electrophoresis. A) eDNAtot, *wa* and *ta* fraction without discriminating between single stranded (ss) and double stranded (ds) forms. B) discrimination of eDNAss*wa* and eDNAss*ta* forms.


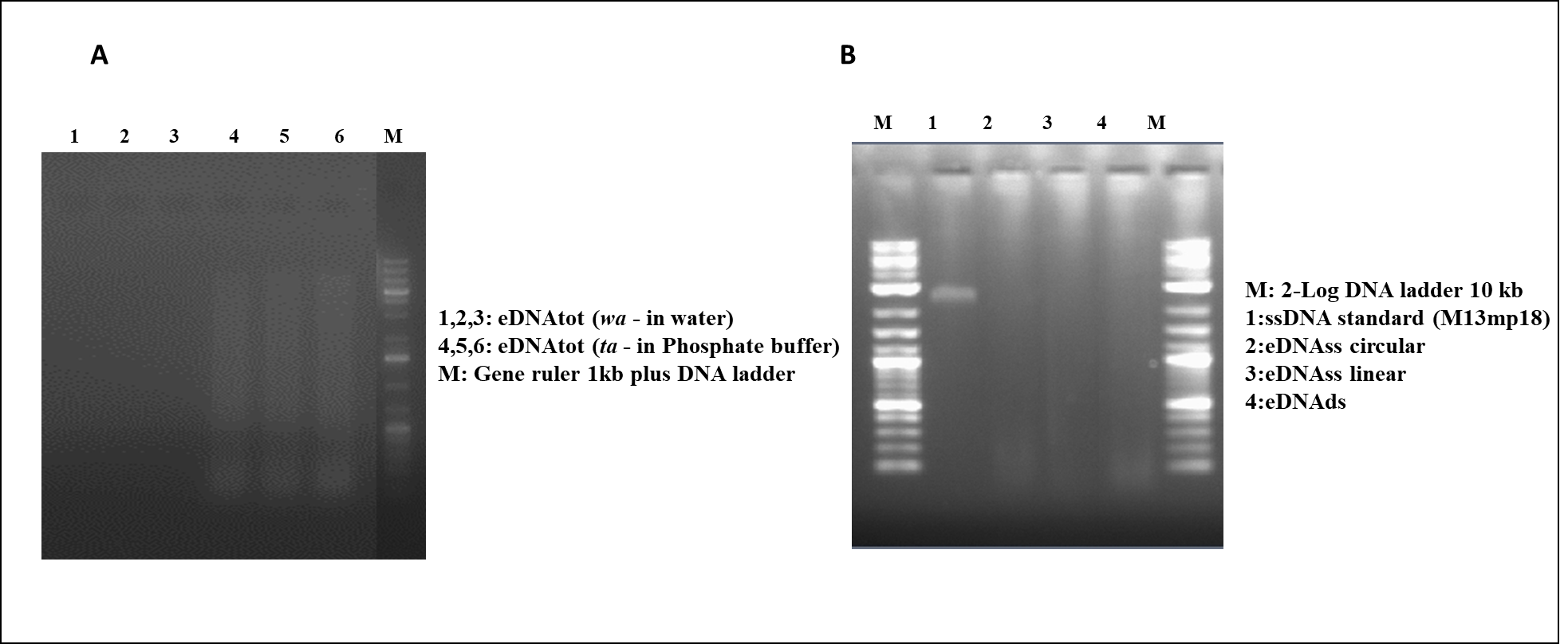

Supplement: S1 Fig — (DOCX) [file pone.0227296.s001.docx]
